# Development of the Everyday Life Rehabilitation model for persons with long-term and complex mental health needs: Preliminary process findings on usefulness and implementation aspects in sheltered and supported housing facilities

**DOI:** 10.3389/fpsyt.2022.954068

**Published:** 2022-08-16

**Authors:** Maria Lindström

**Affiliations:** Department of Community Medicine and Rehabilitation, Umeå University, Umeå, Sweden

**Keywords:** intervention development, implementation, psychiatric rehabilitation, psychosis, accommodations, recovery-oriented, integrated, coordinated

## Abstract

This paper describes the initial phases of the design and development of the Everyday Life Rehabilitation (ELR) intervention, and it presents preliminary findings on usefulness and implementation aspects derived from an ongoing larger trial exploring the effect, cost-effectiveness, and usefulness of ELR. ELR is a model designed to meet the absence of meaningful activities and challenges with integrated, activity- and recovery-oriented rehabilitation in sheltered and supported housing facilities for persons with extensive psychiatric disabilities. The aim of the present study was to examine early experiences of implementing the ELR model from the perspectives of managers, housing staff, and occupational therapists. The paper will sum up preliminary findings based on process data included in an internal pilot prior to a full-scale pragmatic clustered RCT. Four Swedish municipalities with 19 housing facility units were involved and provided process data for the study. Thematic analysis were applied. The informants perceived the methodology to be well suited to the target group and context and to contribute to positive changes in participants' lives. The web-based training was also experienced as relevant and easily accessible to staff, as well as elements of collegial learning and feedback. However, they reported that their municipalities lacked basic conditions for making the whole concept work in such a short time. The ELR is overall perceived as useful, but experiences also make complex difficulties visible regarding integrated, coordinated rehabilitation and organizational readiness. Based on findings, a recommendation on basic prerequisites will be added to the ELR guidance for leadership and management.

**Clinical trial registration:**

[ClinicalTrials.gov, 24 September 2021], identifier [NCT05056415].

## Introduction

Worldwide, health and social care organizations ideally strive to implement models and processes that deliver coordinated, evidence-based, and high-quality care in a professional and cost-effective way (1). However, some health and social care services to certain groups are generally overlooked and underdeveloped, and the amount and quality of efforts differ significantly both within and between countries, and this becomes a health justice issue for already marginalized target groups (2).

When it comes to people with extensive psychiatric disabilities living in sheltered or supported housing facilities in Sweden, these target groups, as well as efforts offered within municipal organizations, usually have a low status compared to other target groups and workplaces. They typically have a low-priority budget, lack of coordinated efforts between different professions, neglected competence supply, and a burdened management (3). Many problems related to health and social care are extremely complex, and involve many actors from different organizational levels that are also subject to different legislations and have different assignments (1, 4). Further, the target groups who have low autonomy, low motivation, and sedentary lifestyle seldom actively seeks out and demands the interventions they are entitled to (5), even though they can benefit greatly from rehabilitation and progress in recovery. Nor do a majority of the housing organizations in Sweden offer integrated, long-term, time-set, and coordinated rehabilitation processes despite the statutory obligation for the care provider to know the target group's needs, suffering, and impact on quality of life (4). This is necessary in order to prioritize (6) and suggest methods and programs for professionals to use in the care processes of discovering and assessing needs and offering person-centered, coordinated, and cost-effective interventions (4).

People who live in sheltered or supported housing facilities (7, 8) must be ensured that they receive the support and health care they need when needed, including rehabilitation (4). However, persons with psychiatric disabilities who live in sheltered or supported housing facilities, often lead a sedentary, lonely life indoors with a downward spiral of few meaningful activities, deteriorating health (9, 10), reduced motivation (5), and further reduced autonomy (11). Thus, there is a need for extended models to improve activity and participation and to support personal recovery and social inclusion for those with the most long-term and complex needs.

There has been a growing amount of research on personal recovery over the past decades, with strong evidence in favor of a recovery approach (12–18), and also on recovery interventions in supported accommodations (19). There is, however, limited evidence to suggest a consistent effect of recovery training for staff (20), recommending that training packages must be adapted to the unique contextual features of these services (20, 21).

### Design and development of the ELR model–elevating everyday life

Given the inequity of the target group and the scarcity of collaborative rehabilitative methods, the ELR intervention model was designed and developed to meet the absence of meaningful everyday life activities, and challenges with, integrated, long-term recovery- and activity-oriented rehabilitation for persons with extensive psychiatric disabilities living in sheltered and supported housing facilities (22, 23).

**Figure 1:**
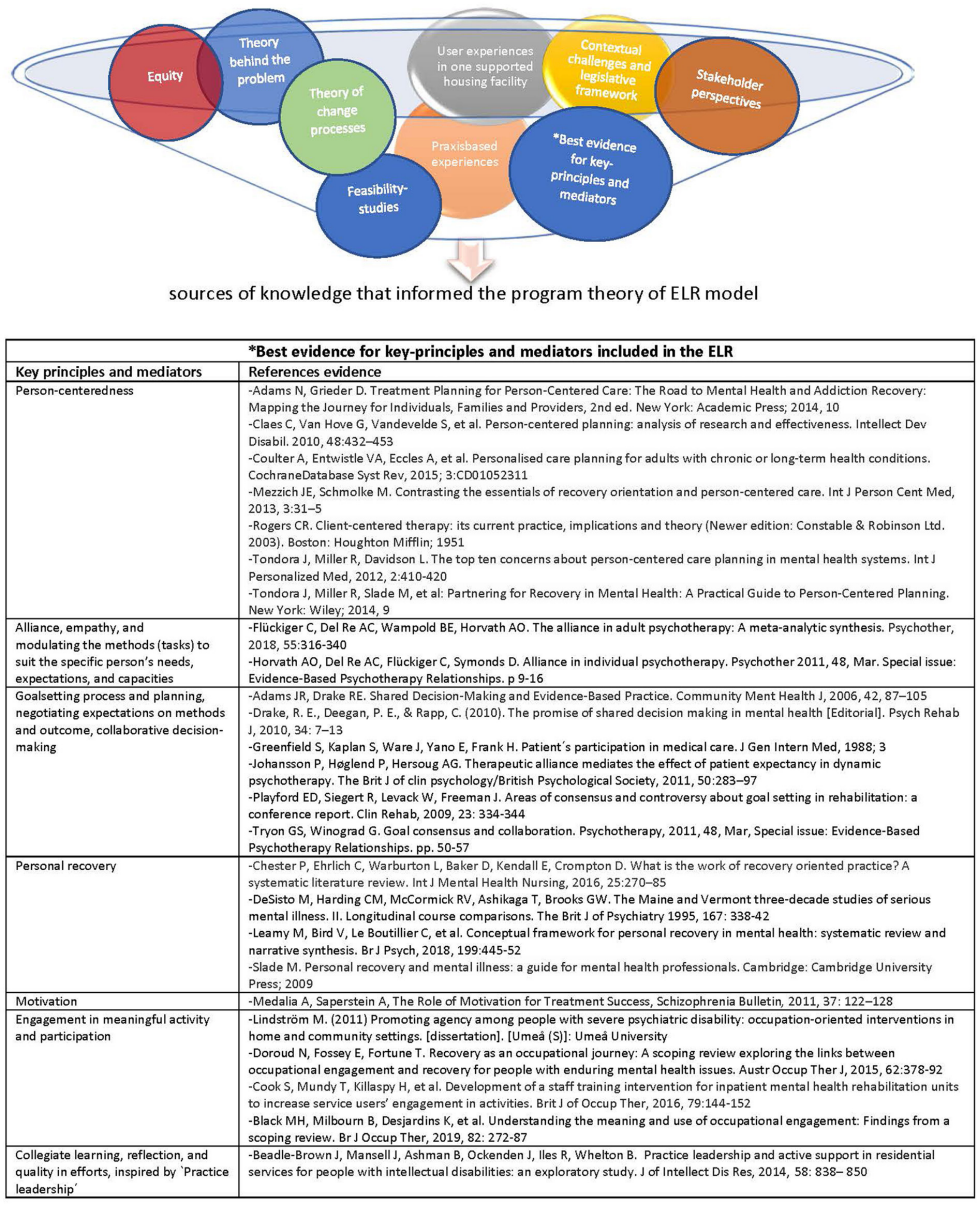
Iterative process of the ELR design and development: sources of knowledge, key principles and mediators, and evidence that informed the program theory of ELR model.

In the ELR model (22–24), focusing on recovery through increased engagement in meaningful everyday life activities is at the center, see Figure 1. Finding enriching activities of the participant's choice and preferences, and exploring and training in real-life situations, is crucial in turning a passive, solitary, and sedentary life into a recovery path with a sense of self-value, belonging, and becoming as a person with individual identity and agency (25).

The design and development of ELR started as an iterative process including practice-based experiences, best available evidence for certain mediators, and consideration for user experiences as well as contextual challenges (22, 23), see Figure 1. The intervention development, and program theory of ELR, is described in detail elsewhere (24, 26).

ELR has been developed in two phases, including (A) a 5-year design and feasibility outcome phase (23) with quantitative and qualitative studies from the perspective of residents, housing staff (HS), and occupational therapists (OTs), see Table 1, and (B) the ongoing pragmatic cluster-RCT involving process studies, whereof this paper synthesizes early process data from the first-year internal pilot study.

**Table 1:** Feasibility outcomes and modifications of the ELR intervention model.

**Impact evaluation of context and intervention**			
**Study and focus**	**Changes in participants (residents)**	**Factors of importance**	**Modifications of ELR**
I. Preparatory study: User experiences of rehabilitation and activity changes in a supported housing facility context–the significance of home	**Personal experiences of:** Taking agentic influence on everyday life activities; making change; increased motivation; easier to get things done; easier to get out of the apartment; increasingly social engagement and social competence	**The supported housing facility context-trying interactions:** Understanding and constructing frameworks; being forced to socialize; being promoted; facing challenges; change leads to further changes (doing change) **- Progressive tensions:** i.e., authentic or artificial; difficult but meaningful; with or without staff	-Emphasizing long-term rehabilitation and support perspectives, using authentic and meaningful real-life challenges, using progressive tensions, and working with inner as well as outer motivation and sociality
II. Outcome of ELR on activity and health variables	**Significant changes in goal attainment, activity- and health-related factors:** Goal attainment; ability; satisfaction; symptoms; effort in activities, i.e., walking in neighborhood, taking part in conversation, phoning, managing hygiene, shopping, cooking, cleaning.	**ELR with its' core principles:** -Collaboration between participant (resident), OT and HS -Partnering person-centeredness -Recovery focus -Activity-based exploration and training in real life settings -Goalsetting of resident wishes -Long-term timed rehab-plan -Continued support during anchoring (maintenance) phase	(Supporting continued research. Modifying research outcome to *recovering quality of life*.)
III. Meaning-making of activity changes in a life context	**Rediscovering personal agency, recovery and positive identity:** Activity and identity changes in a life-history as well as personal/societal perspective **Key storylines:** Emergence of hope in a sedentary life; the dependency of support from staff; re-entering the majority world; the extended value of reaching personal goals	**Mechanisms enhancing rediscovering personal agency and identity:** -Infusing hope -Transparency in process and attunement to the individual -Doing transformations -Agent-supported rehabilitation -Out-of-housing strategies -Extended value of reaching goal/thorough goal setting process	-Underscoring social arenas and continuously deepened connectedness, using reflection on meaning and the doing, and strengthening sense of identity and personal agency
IV. Involvement and experiences of housing staff	**Varying staff experiences regarding participant's change:** -None: have not met the resident and not thought of it -Temporary: happy and engaged in doing but will probably not last -Sustained gains: attained specific goal area and continues to pursue it -Further gains: positive side-effects in other activity areas, joy, pride, self-directedness, increased social life	**Disability ideology:** Disparity between stabilization/recovery **Staff and organizational attitude:** Resistance/responsiveness **Shared framework:** -Enhancing collaboration -Help focusing the right things -Using person-centeredness -Recovery- and activity focus -Promoting person-driven goals	-Converting education package to web-based training for HS and OTs -Clarifying the tools for collaboration -Clarifying management and leadership -Adding monthly follow-up by HMs, for collegiate learning and reflection
V. OT experiences of complex processes	**Positive changes as experienced by OTs:** Negative spirals turned into positive with signs of positive energy and willingness to establish contact and change in everyday life, gained insight and understanding of oneself, expressing personal preferences, increased motivation, sense of worth, increased activity, having friends, successful goal attainment and rehabilitation processes, restored power to effect change in one's life	**Supporting personal recovery and activity-important events and interactions that shape complex therapeutic processes**: A preparation phase for both; building companionship and trust; uncovering wishes and agreeing on expectations for goals and methods; understanding and upholding fluctuating motivation; achieving changes in everyday life; using actions as insightful events; and opening the door to society and new arenas	-Clarifying transparency and negotiation of expectations -Adding a preparation phase to the change- and anchoring phase -Emphasizing validation, confirmation, and reflection in combination with the doing

I. Lindström et al. (27). II. Lindström et al. (28). III. Lindström et al. (25). IV. Lindström et al. (29). V. Lindström et al. (30).

Overall, the ELR intervention model has been well received by OTs (30), as well as by residents (25, 28) indicating very promising tendencies, such as successful rehabilitation with goal-attainment, health, and re-engagement in activities. Among HS, the ones who have embraced ELR and a collaborative approach have found ELR to be very helpful and encouraging both for HS and for residents, while the ones who have been resistant toward a new method and avoided collaboration have found ELR to be a waste of time and resources (29). Feasibility results suggest that ELR reduces institutionalized patterns and helps turn a negative spiral into a positive spiral with out-of-the-house activities, social participation, and overall increased engagement in enrichening activities among residents (23).

Previous findings from our feasibility studies of ELR have resulted in minor modifications to the intervention prior to the RCT, such as converting the educational package into a web-based training for HS and OTs including reflections, and adding monthly follow-up inspired by “practice leadership” (31), focusing on quality, collective learning, feedback, and coaching.

### Rationale for the study

There is a need for evidence-based interventions that are useful and acceptable. ELR has been shown to be a promising intervention that involves HS, OTs, and residents in collaborative, activity- and recovery-oriented rehabilitation. As such, ELR merits further evaluation to establish its effect, cost-effectiveness, and usefulness, as well as crucial implementation aspects.

The aim of the present study is to examine early experiences of implementing the ELR model from the perspectives of housing managers (HMs), HS, and OTs.

## Materials and methods

The current project's first wave, year one, was designed as an internal pilot partly to calculate the effect size and relevant numbers of participants and partly to capture implementation aspects at an early stage in order to detect any need for revisions or clarifications prior to the full-scale RCT including long-term process research. Thus, the method and data for this brief research report are but one part of several ongoing studies included in the ELR trial.

Thematic analysis (32) and synthesis of process data were applied for this study, focusing on perceived usefulness and implementation aspects during the first-year internal pilot.

### Intervention model

**Figure 2:**
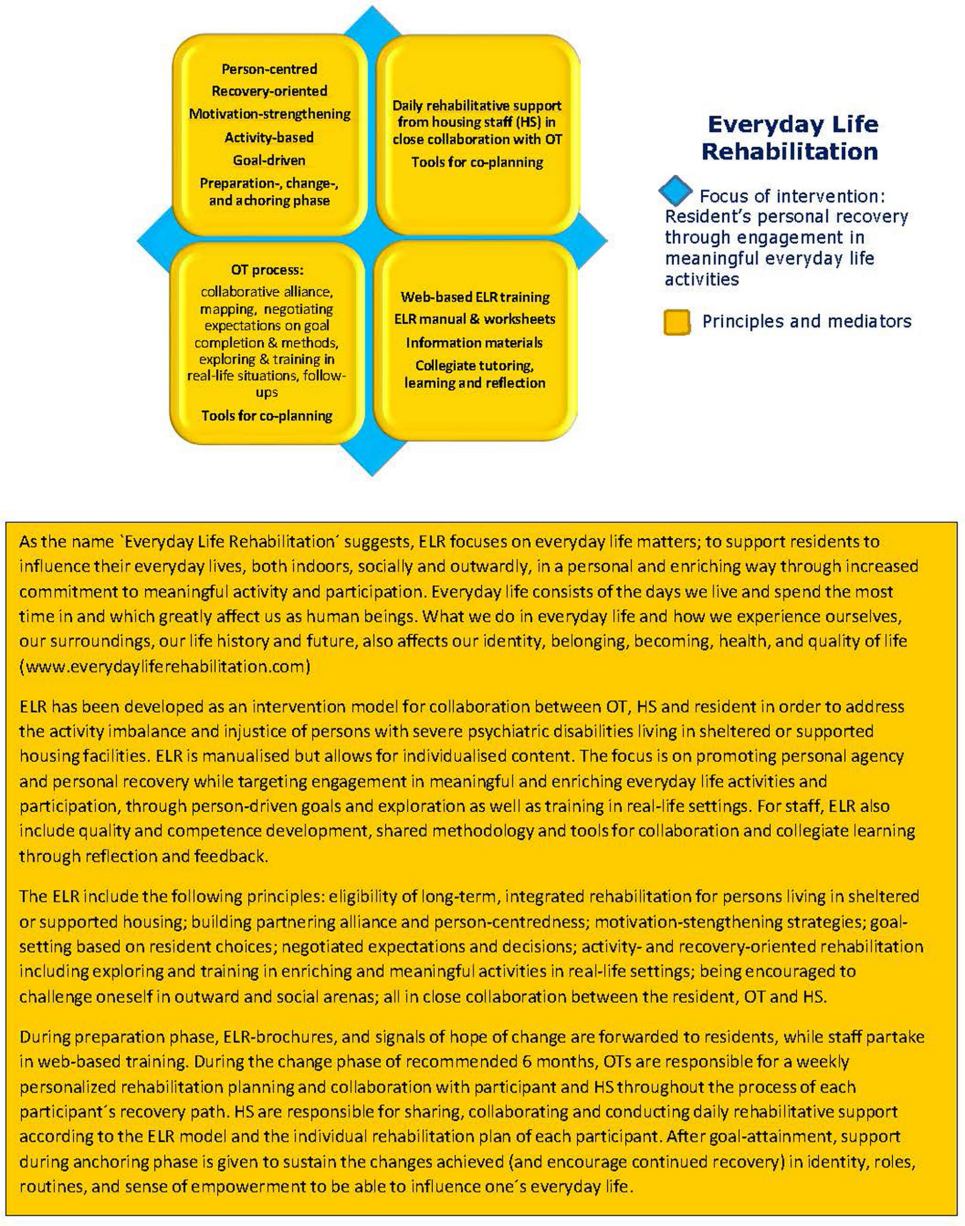
The Everyday Life Rehabilitation (ELR) intervention model.

ELR includes training, methodology, and tools aimed at HS, OTs, and HMs so that they in turn can collaboratively offer and implement person-centered, motivation-strengthening, activity- and recovery-oriented rehabilitation to people with severe psychiatric disabilities living in housing facilities (24), see Figure 2.

### Context and participating municipality housing units

Four municipalities in northern Sweden, with varying numbers of housing units within each municipality, participated during year one. Municipality A had only one housing unit, municipality B had three, municipality C had seven, and municipality D had eight housing units that participated in the ELR internal pilot. The size of the accommodations varied between 3 and 14 residents per housing unit, and the number of HS employed in each housing unit varied depending on the number of residents and their needs. A total of 12 HMs were responsible for the 19 total housing units, and these were mainly stationed in another location close to the municipality's management center.

Seven experienced OTs were connected to the 19 housing units within the four municipalities. The OTs were employed within a municipal health and medical care organization but were commissioned to collaborate with the HS, see Table 2.

**Table 2:** Contextual features of sheltered and supported housing in Sweden, and concepts used.

The role of the municipality, and legislative demands	In Sweden, people with severe and long-term psychiatric disabilities are entitled to live in sheltered or supported housing facilities, so called ‘LSS or SoL housing' according to the Act concerning Support and Service for Persons with Certain Functional Impairments (SFS 1993:387, LSS) or the Social Services Act (SFS 2001:453, SoL), when the disability is causing significant difficulties in daily life and thus requires extensive support or service by HS. It is mandatory to also offer basic healthcare, including rehabilitation, within the housing facilities (SFS 2017:30, HSL), and must be documented in an individual plan/rehab-plan. Thus, there are two areas of municipal responsibility where professionals, in order to meet the legal requirements, must collaborate in their work with the respective residents. (Only a few municipalities in Sweden offer interventions in line with the legal requirements, thus reflecting unequal care and rehabilitation.)
Sheltered and supported housing facilities	These terms describe two different forms of accommodations organized by the municipality. Here, ‘sheltered housing' refers to 24-h staffing and time-unlimited support to residents. ‘Supported housing' refers to staff based onsite up to 24-h per day. The number of people living in specific housing units can vary greatly but the type of support is similar.
Rehabilitation planning	‘Rehabilitation' and ‘recovery' might be interchangeable, and both concepts can refer to either the process-steps led by professionals or the personal process of regaining meaningful activities, social roles etc. Here, the ‘rehabilitation planning' or individual rehab-plan, refers to the commitment of a registered OT, who is responsible for mapping, assessing needs, negotiating goals and methods, leading and following up a participant's change process toward their individually set goal. Recovery-oriented rehabilitation include support to develop increasing connectedness, hope, identity, meaning, and empowerment (CHIME) ^(*)^, but are not mandatory in Sweden.
Personal recovery path	Refers to the personal process of building a meaningful and satisfying everyday life of wellness and participation in society, despite severe mental illness and symptoms. In synthesized research, the five-domains of the CHIME model ^(*)^ have been crystallized as common aspects, that is the personal process of increasing connectedness, hope, identity, meaning, and empowerment.
HS roles and responsibilities	HS have a responsibility to support residents in their everyday lives. The methods are usually not regulated, and the level of competence is generally low ^(**)^. However, potential in the assignment exists in the form of acquiring methods and developing collaboration with healthcare rehabilitation efforts, such as recovery and activity-oriented rehabilitation based on the person's own wishes (in line with the ELR).
OTs roles and responsibilities	OTs have responsibility for assessing needs and planning individual rehabilitation, as well as collaborating with HS. OTs are responsible for full documentation according to statutory patient records. OTs are not stationed in housing, and belong to an organizational affiliation other than HS, whereupon ‘treatment as usual' in many municipalities have been described as single, short-term initiatives at the request of housing staff, such as prescribing cognitive technical aids. However, potential in the assignment exists in the form of outreach and motivation-strengthening offers of long-term recovery and activity-oriented rehabilitation based on the person's own wishes (in line with the ELR).

(*) Leamy et al. (16).

(**) Socialstyrelsen (33).

### Implementation structure and responsibility

The operations managers were primarily responsible for decisions on the introduction of ELR and for the overall implementation in practice.

Each participating municipality appointed a contact person for contact and planning between the municipality and the research group.

Adherence to the time and implementation plan for the ELR model and trial was the responsibility of the local HMs of each housing unit in the participating municipalities and of the health division managers for the OTs. HMs and health division managers were also responsible for ensuring that HS as well as OTs took part in the web-based educational package and participated in the monthly follow-ups focusing on collective learning, reflection, feedback, and coaching. Further, the HMs and health division managers were responsible for the collaborative structure of the initiative by enabling the use of the ELR tools for collaboration.

OTs and HS were responsible for adherence to the ELR model, in guiding participants in their rehabilitation plan and personal recovery path.

### Data generation

Five different qualitative sources of data generation were included in this study:

1. Management representatives were initially asked to describe “treatment as usual,” to describe the challenges, needs, and overall ambitions with efforts for the target group, and to describe their future vision for introducing a new method.
2. HMs were asked to document, on a monthly basis, a summary of the views obtained from both HS and their own reflections on how the ELR introduction and implementation proceeded.
3. OTs were responsible for a process diary and a research protocol as requested for the trial.
4. A validated web-survey with both closed (Likert-scale answer alternatives) and open-ended questions was distributed to all involved HS. *Only the open-ended qualitative part was included in this study*.
5. Similarly, a validated web-survey with closed and open-ended questions was distributed to all involved HMs. *Only the open-ended qualitative part was included in this study*.

The first author (ML) instructed and obtained data from sources 1–3, CK obtained data from source 4, and MH obtained data from source 5.

### Data extraction, synthesis, and thematic analysis

A thematic analysis (32) was conducted by ML. Each source of data was first read repeatedly to become familiar with the material, then initial codes were generated based on informants' perceived usefulness and implementation aspects. Codes from the different sources were compared, examining similarities and differences, and then organized and synthesized into overarching themes before reviewing the preliminary themes, see Table 3. The codes and preliminary themes were discussed with the study team for process research in order to critically review the interpretations in relation to the raw data, and minor modifications were made.

**Table 3:** Example of codes and related theme.

**Example of codes**	**Related theme**
Well-functioning for residents and staff but time-consuming, takes time to fully implement	Perceived usefulness of ELR
Based on collaboration and quality to users, at the same time competence development and collegial learning for staff	
Ideal way of working, increased collaboration	
Benefits for residents becoming more active	
Users having a more eventful life with more impressions and personal value	
Helpful for staff focusing on the right thing	
Basically good but needs to be simplified	
Nice way of working, educational, great to collaborate	
ELR make us do a better job, would like to continue with more users	

## Results

The synthesized and overarching themes are presented below. In order to enhance trustworthiness, direct quotations (in italics) from all data sources are presented in relation to the themes constituting the results.

### Municipalities' starting point and future vision of introducing a new method

The management representatives expressed a lack of evidence-based methods in their housing facilities, and thus they had an interest in introducing a model based on the best available evidence for several mediators and principles and relevant to their particular context. A couple of municipalities emphasized that the design in ELR felt adapted to precisely these contexts and that it was important that it was not an overly burdensome effort. Management also expressed a need for investment after a stressful pandemic, both for staff and residents. Above all, they described the need for skills development and valued that ELR offered easily accessible and free of charge web training with sections that could be completed at the individual's own pace and that could be worked on in calm work situations.

*“We value the idea of a joint investment in competence development and a common methodology for housing staff and rehab staff, especially the motivation-strengthening methodology, but also the person-centered and recovery-oriented focus and the whole of ELR, with increased activity and meaningfulness for residents.”*

Management representatives stated a great need for working methods for both staff and disadvantaged target groups. However, on a specific question about their future vision for introducing new methodologies, all were vague and did not express anything specific.

### Adherence to the time and implementation plan for the ELR model and trial

According to the time-, implementation-, and trial-plan, ELR brochures were meant to be distributed before summer to all residents with psychiatric disabilities so that they could think over the summer about whether they wanted to participate in an activity and recovery-oriented rehabilitation period of 6 months based on something they themselves wanted to develop in their everyday lives. However, it turned out that in some housing units it was HS who directed the invitation only to a single individual whom they thought could benefit from ELR.

During the ongoing implementation, a couple of HMs changed workplaces, and as a result information to HS in some housing units was missed. HS were not informed that a new method would be introduced, nor that they would take part in web-training for a 2-week period.

Among both HMs and HS who had applied monthly follow-up reflections proposed within the ELR guidelines, they expressed positive experiences from feedback and sharing examples. There was, however, a mixed response rate lacking some follow-up occasions, indicating that the monthly follow-up was not applied as frequently and as continuously as intended.

*“It's fun when the HM shows interest in how we work but could be more of it, then you feel noticed. It makes us talk more about how we work and learn from each other.”*

### Educational package

OTs and HS generally perceived the web-training as good, with clear and relevant content. Some elements, such as person-centeredness, were partly perceived as repetition, while much was new. The accompanying worksheets were perceived as somewhat too difficult with partly too academic language. The format was appreciated as flexible and easily accessible:

*“Nice to be able to watch the episodes at any time, when it fit into the work, when a quiet moment was available*.”

The associated reflection questions for each section were also experienced as providing added personal insights and thoughts about practical application, as well as contributing to value and importance:

*“It also gave a guiding signal that this would be followed up by the HM, which helped to really get into this and write down personal thoughts.”*

Also, among the HMs who chose to take part in the web-training, they expressed that it was positive and important:

*“I thought it was good to take part in the web-training as a manager because I could ask the follow-up questions and be responsible for the HS's work.”*

### Collaborative commitment at all levels for applicable implementation in practice

After the staff's training, a rehabilitation period began for participants who had accepted the invitation. Several voices expressed that when ELR was used as intended it worked very well in most cases, while it was difficult and burdensome when collaboration did not work. Regular collaborative meetings were found to be important.

The HS who were not informed or who had not participated in the web-training were not prepared to cooperate as intended. In addition, other reasons were also mentioned by HMs, as to why some HS were negative and did not want to partake in the web-training, such as resistance to introducing new methods, reluctance to cooperate on rehabilitation, and opinions that the residents are not capable of participating in a rehabilitation process. These views were accepted, without action or pressure, which meant that some housing units consistently had a negative attitude toward the participants' rehabilitation process. A couple of participants explicitly refused to involve HS because they had recurring condescending comments, for example, “*You walk such short laps, this cannot improve your health.”*

In contrast, within the housing units where HS initially participated in the web training and also in meetings about the forms of collaboration based on ELR, positive signals emerged. They were described by OTs as much more involved in the continued collaboration on the rehabilitation of individual participants and were willing to share input. HS also expressed positive experiences of HMs informing, collaborating, and following up on how things went along the way and that they were able to share information with each other. Further, the HS described advantages of applying the same methods:

*“Benefits, clearly, to work with the same methodology between OT and us; it upgrades the status, becomes more fun to work, easier to understand problems, adds clearer plans and tools both for treatment, motivation and to attract resident's own will.”*

Both OTs and HS also expressed positive experiences of learning collaboratively and incorporating collaborative action planning with meaningful activity and recovery into the rehab path:

*“We try to learn from each other how to bring activity and CHIME into the rehab-process, and I think it deepens the personal change in participants; to constantly give hope, support motivation by eliciting one's own will; to guide the person to discover old or new arenas and provide support for deepened connectedness; to talk about meaning and the doing; to work with their sense of identity as something else than their illness; and self-efficacy to be able to influence one's everyday life with elements of activity and sociality.”*

### Perceived usefulness of ELR

ELR was overall perceived as a well-functioning and suitable method for the target group and context by OTs, HS, and some HMs. HS expressed that it was impressive to see how deep an understanding of a participant the OTs developed through conversations and different methods and how they managed to elicit desire and motivation in the activity. The OTs also expressed positive experiences:

*“It feels like an ideal way of working. This is how we truly want to work, with long-term rehab plans for participants and with good collaboration between the participant, HS, and us.”*

HS described challenges of working according to ELR in terms of time and requirements: “*time-consuming to learn new things, and being allowed to take time to work with a resident outside the home, for example getting out into nature.”*

Positive voices from HS described ELR as a complex but useful method with benefits such as users becoming more active, outgoing, having a more eventful life with more impressions and personal value, strengthening their wellbeing, being encouraged to influence and enrich everyday life despite bad mood, and receiving positive feedback thanks to the frequent reflections. In one exemplified residence (see Appendix), both the manager and HS sang the praises of the collaboration and what it contributed to the participants. HS were able to practically and gradually support the participant and found it fun to be so involved in a change process in interaction with the OT and the participant:

*“We developed a plan, which took shape based on the input of both the user, us, and the OT. The user had a fairly advanced goal, but we planned a gradual approach and took advantage of shared knowledge, and new angles along the way. It was structured but still flexible, leading to many positive approaches and steps of development.”*

### Organizational readiness and conditions

Municipalities' readiness to introduce and implement a new method was described as insufficient by informants from several of the sources included. Similarly, ambiguities emerged in expectations for municipalities' internal processes, with its unclear signals and lack of commitment from management, in relation to the role of the research team. More specified control documents with time indications for management have been requested by HMs and OTs.

Initially, it turned out that basic, statutory routines for collaboration concerning rehabilitation and self-care processes were missing or unclear within the municipalities in question. The OTs described how it took extra time and energy from them initially, effort for which they are not really responsible. It also took extra time for OTs to inform and include HS in the housing units where information was missed.

Outspoken expectations from management along with established routines for rehabilitation and collaboration were mentioned as necessary prerequisites for the startup, as “*ELR became a voluntary choice among both HMs and HS*.” The lack of long-term vision and lack of processes for implementing a new method in the municipality, together with a lack of outspoken expectations from management and a lack of established routines for rehabilitation and collaboration, were identified as the main contextual barriers.

“Practice leadership” was perceived favorably, with the elements of collegial learning, feedback, and a focus on quality in the efforts of supporting participants' recovery. However, the HMs were in general overloaded with administrational tasks and had difficulties implementing the monthly follow ups.

## Discussion

This study was part of the first-year internal pilot of ELR prior to the full RCT including long-term comprehensive process studies. The main purpose was to evaluate implementation aspects at an early stage in order to decide upon continued actions. Overall, ELR was perceived as useful, but experiences also made complex difficulties visible regarding integrated, coordinated rehabilitation and organizational readiness.

The results show both facilitating aspects and barriers to implementing ELR. ELR was overall perceived as a well-functioning and suitable method for the target group and context. Facilitating factors included the outspoken need of competence development, evidence-based methods, easily accessible web training, and appreciation of a shared methodology in collaboration between participants, HS, and OTs. Another facilitating aspect was the use of monthly follow-ups by HMs regarding HS's reflections and practical examples inspired by “practice leadership” (31) and focusing on quality in the efforts with the participants.

Contextual barriers were found to be the lack of municipal long-term vision and lack of processes for implementing a new method, together with a lack of outspoken expectations from management and a lack of established routines for rehabilitation and collaboration. Confidence in residents' capacity to participate in ELR was generally low, and in many housing units HS decided whom to invite instead of it being the resident's choice. Another barrier concerned the introductive, follow up, and coaching capacity of HMs. In housing units where HMs informed and prepared the HS, and they could take part in the web-training, the ELR was well received.

These results are in some respects similar to several implementation studies of new methodologies in municipal activities (34, 35). A recurring result is the shortage of organizational readiness. Also, that unit managers are heavily burdened (3) and thus do not have time to work with quality and methodology. The consequence of an administrative and distant leadership can be unclear norms and voluntary choices of methods, attitudes, and approaches in some housing facilities (31). Thus, the conditions that public sector managers work under need to be addressed (3), as well as fostering a cultural change. Given that health and social care systems are complex and adaptive, it is extremely challenging to make organizational improvements (36). Large-scale change initiatives, such as comprehensive policies, have been proposed to address problems in health and social care systems (1).

In activity- and recovery-oriented rehabilitation, increasing a person's engagement in daily activities has been found to enhance health and recovery (23, 37–39). Internationally, psychiatric rehabilitation services have increasingly embraced and implemented a recovery-oriented approach (12–19), and in many countries, a recovery focus is mandatory based on its strong evidence and well-documented benefits.

### Methodological considerations

The data set was based on two small and two middle-sized municipalities. Data from some housing units were incomplete or completely missing, and the limited selection and short time perspective means that the results must be considered preliminary.

ML is an assistant professor in occupational therapy, and is the original developer of ELR and is the primary investigator for the full-scale project. As such, there is a potential risk to interpret results more positively and in favor of further development of ELR. At the same time, it is of shared interest to capture perceived difficulties at an early stage regarding both the intervention and its implementation in order to be able to revise or clarify the intervention and to conclude whether the intervention is working ethically, practically, and structurally.

These risks and benefits have been discussed from an ethical perspective and have been discussed openly within the research group in order to regularly reflect on and be vigilant about such risks. There is no financial gain involved, but rather a health justice pathos regarding access to high-quality rehabilitation, recovery, and increased quality of life for persons with psychiatric disabilities. CK is PhD and researcher in health sciences, and MH holds a position as a development coordinator for the disability area.

## Data availability statement

The datasets presented in this article are not readily available because they include sensitive data, involving risk of identifiable data, which is why restrictions apply to the dataset. Requests to access the datasets should be directed to Data Management Plan (DMP) for the Everyday Life Rehabilitation Project (dmp.umu.se; ID 86475).

## Ethics statement

The studies involving human participants were reviewed and approved by Swedish Ethical Review Authority (Dnr 2020-06220). The patients/participants provided their written informed consent to participate in this study.

## Author contributions

ML is the Principal Investigator for the trial, author of this brief research report, responsible for the design and development of the ELR, and she generated the idea for the trial and led the proposal.

## Funding

This trial was funded by the Swedish Research Council for Health, Working Life and Welfare (FORTE 2021-01391). The funder does not have a role in study design, analyses, manuscript, or in the decision for publication.

## Conflict of interest

The author declares that the research was conducted in the absence of any commercial or financial relationships that could be construed as a potential conflict of interest.

## Publisher's note

All claims expressed in this article are solely those of the authors and do not necessarily represent those of their affiliated organizations, or those of the publisher, the editors and the reviewers. Any product that may be evaluated in this article, or claim that may be made by its manufacturer, is not guaranteed or endorsed by the publisher.

## Abbreviations

CHIME: Connectedness, Hope, Identity, Meaning, Empowerment
COPM: Canadian Occupational Performance Measure
ELR: Everyday Life Rehabilitation
HM: Housing Manager
HS: Housing Staff
RCT: Randomized Controlled Trial
OT: Occupational Therapist.
