# Supplementary material for: Development of the Everyday Life Rehabilitation model for persons with long-term and complex mental health needs: Preliminary process findings on usefulness and implementation aspects in sheltered and supported housing facilities
Source: Front Psychiatry. 2022 Aug 16;13:954068. doi: 10.3389/fpsyt.2022.954068 (PMC9424656; doi:10.3389/fpsyt.2022.954068)
Supplement: Supplementary file 1 [file Data_Sheet_1.PDF]

Appendix: Case description (data-sheet revised not to reveal identity or context)

One example of a participant´s rehabilitation- and recovery path during 6 months time-set integrated rehabilitation, guided by ELR

Preparation phase

During summer, ELR-brochures were distributed to the participating housing facilities. Potential participants had the opportunity to consider whether they wanted to agree to a rehabilitation period of six months, with an approach and focus based on ELR. Kim chose to grab the chance and participate.

Thereafter, housing staff (HS) encouraged Kim in choosing to participate in ELR and the hope of possible change in enrichening everyday life and recovering quality of life. HS handed out a worksheet with preparatory questions to participants, prior to the start. Kim pondered upon how everyday life ideally would look like, and what was most inspiring to enrichen everyday life with, or find a better daily functioning in.

(Meanwhile, HS and OTs participated in a 10-section web-based training, with an accompanying ELR-manual, and tools for collaboration, before ELR was started with participants. Housing Managers (HM) took part of a brief manual inspired by practice leadership, and two monthly follow up questions.)

Change phase

After the staff´s training, a rehabilitation period began for Kim. Kim was nervous but expectant at the first meeting with OT.

OT described how a rehab process usually takes place in different steps from getting to know each other, through conversations and mapping, to choosing your own goal, with negotiation of expectations about desired end results based on goal picture and possible methods to get there, then exploration and training in activity in selected environments, with a gradually increasing challenge to finally reach the goal, as well as follow-up both along the way and at the end.

Kim told about the worksheet that aroused some thoughts, including whether it would be worth investing in a fun leisure activity or trying to find better routines around showering, which felt pressing and difficult but important. OT suggested using COPM next time to review what an ordinary day looks like today and what feels most important to prioritize for a rehab period.

Kim said hobbies had completely died out and it felt difficult to choose someone. OT then suggested that Kim for the next time could check out a list of leisure activities that felt enticing and something to explore together in the forthcoming period.

Next meeting began with looking at the checklist of hobbies. Kim had chosen dance, which felt enticing but completely new. OT encouraged and said dance was also an activity that over time could be developed in social contexts. Then COPM was applied, which resulted in Kim choosing the leisure activity dance as a top priority, but that it could also be necessary to be clean and smell good, why Kim also wanted to set challenges around showering routines.

OT consulted with HS about their picture of Kim´s problems with showering. They told everything had been tried over the years but nothing worked. They tried to urge Kim but it rarely led to a shower. OT wondered what the cause of the problem was but no one knew. OT then wondered if it could be due to sensory problems, or psychotic thoughts, motivation, or something else? Come to reason with Kim about this in order to approach an understanding of cause and thus appropriate action strategies.

OT suggested Kim to fill in a sensory profile, but it did not reveal anything special linked to showering, however sensitivity to light and strong sunshine, which often led to headaches. OT also reasoned with Kim if there were unpleasant thoughts connected to showering, but it was rejected by Kim. Kim thought that it was due to own resistance, it felt so hard to shower, that the initiative was lacking and it felt insurmountable to start the steps around showering.

OT also talked to BP about Kim´s prio to learn to dance, which Kim chose as the main goal. OT revealed that she did not know much about dance but it turned out that two BPs were experienced dancers and were happy to help. Kim wanted to set aside one hour a week for dance training. Together with Kim, BP and OT, a rehab plan was designed, which consisted of telephone support before showering (BP hold talks and guide Kim to the shower), and set aside time once a week with a step-by-step challenge, where available BP would lead the session, with tips from the experienced BP:

- Explore music styles, feel the rhythm, move to music, explore preferences, emotions and flow
- Introduce and try different dance steps (fox)
- Dance fox with experienced BP
- Invite residents from the neighboring house to a dance event
- Participate in dance associations´ try-out opportunities

- Make study visits to social dance events
- Find friends to go dancing with.

The strategy when BP phoned and guided Kim to the shower worked well and was experienced simly by both BP and Kim. When it came to dance, it turned out that complicated step combinations became too difficult and theoretical, while Kim liked simple steps and to express emotion in dance. A dance association with focus on exploratory free-dance became the company that Kim liked best. Kim decided to join the association and could go there self on occasion, but wanted continued support to get started with a shower. Kim also started to join social dance events in fox, together with another resident from the neighboring accommodation. Kim reached the goal above the expected level.

During the ongoing rehab-period, both BP and OT repeatedly talked to Kim about recovery-inspired dimensions, such as connectedness, hope, identity, meaning, and self-efficacy to be able to influence one´s own everyday life in a positive way. Kim was especially pleased to have found a fun leisure activity that also gave a new identity as a dancer and new social arenas in life.

Anchoring phase

To ensure that Kim would receive continued support to maintain dance and showering, OT made a self-care decision, with a self-care plan that was transferred to the LSS journal under `support for self-care´. The plan included continued strategies for BP to call and guide Kim to the shower before the dance, and to encourage Kim to maintain commitment to the dance association with free-dance, as well as social dance fox, by talking about and keeping an eye out for upcoming dance events. Further, to encourage Kim to continue developing towards recovering quality of life.
